# Genetic Determinants of Milk Production Phenotypes in Dairy Goats: A Review

**DOI:** 10.3390/vetsci13080799

**Published:** 2026-08-13

**Authors:** Shuaishuai Wu, Mohamed Tharwat, Abd Ullah, Nourhan Nassar, Abdulrahman A. Alkheraif, Muhammad Zahoor Khan

**Affiliations:** 1 College of Animal Science and Technology, Henan University of Animal Husbandry and Economy, Zhengzhou 450046, China; 2 Department of Clinical Sciences, College of Veterinary Medicine, Qassim University, P.O. Box 6622, Buraidah 51452, Saudi Arabia; atieh@qu.edu.sa; 3 College of Agriculture and Biology, Liaocheng University, Liaocheng 252000, Chinazahoorkhan@lcu.edu.cn (M.Z.K.); 4 College of Veterinary Medicine, Huazhong Agricultural University, Wuhan 430070, China; 5 Department of Pathology and Laboratory Diagnosis, College of Veterinary Medicine, Qassim University, P.O. Box 6622, Buraidah 51452, Saudi Arabia

**Keywords:** dairy goat, milk production traits, candidate genes, casein gene cluster, marker-assisted selection, GWAS, transcriptomics

## Abstract

Dairy goats sustain milk production across marginal environments worldwide, yet genetic improvement of milk yield and composition remains slower than in cattle, constrained by smaller reference populations and limited functional validation. This review consolidates current evidence on the genes and polymorphisms governing milk production traits in dairy goats. Whole-genome resequencing and selection-signature scans repeatedly recover a lipogenic–somatotropic core (*ACSS2*, *DGAT2*, *GHR*, *VPS13C*, *PRPF6*), whereas genome-wide association studies (GWASs) confirm the casein cluster (*CSN1S1*, *CSN1S2*, *CSN2*, *CSN3*) as the most reproducible determinant of protein content and *DGAT1* as the principal fat-content gene. Transcriptomic, single-cell, and metabolomic analyses, together with functional dissection in goat mammary epithelial cells, resolve the regulatory networks—*SREBP1*, *PPARG*, *ELF5*, and non-coding RNAs—that translate genotype into phenotype. Integrating multi-omic evidence with expanded, well-phenotyped populations is essential to convert this expanding catalog into precise marker-assisted and genomic selection for milk yield and quality.

## Simple Summary

Goats are a mainstay of milk production in dry and difficult regions, supporting the livelihoods of millions of smallholder farmers and supplying milk that is nutritious, easy to digest, and less likely to trigger allergies than cow milk. Yet breeding goats for higher and better-quality milk has progressed more slowly than in dairy cattle. Modern DNA-based tools can accelerate this progress by pinpointing the genes that shape how much milk a goat produces and how rich it is in fat and protein. This review brings together findings from many studies to identify which genes most reliably influence these traits and how they act inside the udder. Such knowledge allows breeders to select superior animals earlier and more accurately. Realizing these benefits will require larger, carefully recorded goat populations and the combining of different biological datasets, ultimately helping farmers raise more productive herds and improving the supply of high-quality goat milk.

## 1. Introduction

Dairy goats occupy a strategically important position in global animal agriculture, particularly across Mediterranean, African, Middle Eastern, and Asian regions, where their adaptability to harsh production conditions and climatic variability makes their contribution to marginal areas difficult to match [1]. For smallholders, dairy goat farming provides a tractable route to improved livelihoods through milk production and income generation, with the potential to strengthen food security and reduce poverty [2,3]. The global goat population exceeded 1.1 billion head in 2022, including more than 200 million dairy goats that together yield an estimated 20.7 million tons of milk annually [4]. Their value in arid and semi-arid zones is amplified by an economic logic centered on risk reduction rather than output maximization, which suits the volatile conditions faced by small-scale rural households [1,4].

Goat milk is further distinguished by its nutritional and functional properties [5,6,7,8]. Its fat globules average approximately 3.5 µm in diameter, against roughly 4.5 µm in bovine milk, and the resulting increase in interfacial surface area exposes more substrate to lipases and improves digestibility [9,10]. A high proportion of short- and medium-chain fatty acids reinforces this advantage, while a β-casein/αS1-casein ratio resembling that of human milk and a comparatively low αS1-casein content render goat milk less allergenic than cow milk [11,12]. Together, these attributes have driven growing consumer demand for specialized goat dairy products such as cheese and yogurt [10].

The economically decisive milk production traits—milk yield, fat percentage, protein percentage, and lactose content—are quantitative in nature and are therefore shaped by numerous genetic and environmental factors and their interactions [13,14,15,16]. Conventional improvement based on phenotype and pedigree is effective but slow, especially for traits that are difficult to record or exhibit low heritability. Accordingly, the application of genomics in livestock has advanced from marker-assisted selection toward whole-genome selection, substantially raising the ceiling on attainable genetic gain, and genome-wide association studies (GWAS) together with selection-signal scanning are now routinely used in dairy goats to localize genomic regions and candidate genes underlying milk yield and composition [13]. These traits are highly polygenic and typically governed by many loci of small effect alongside a few of major effect; the major genes so identified can be deployed in marker-assisted selection or used to sharpen the accuracy of genomic evaluation [13,17,18].

Among candidate loci, the casein genes have attracted the most sustained attention. Milk protein genes are the most intensively analyzed class, and major effects have been documented for αS1-casein in both sheep and goats [19]. In particular, polymorphism at the goat αS1-casein (*CSN1S1*) locus exerts a major influence on milk protein, casein, and fat content and on cheese yield [20]. More recent work in the Sarda breed, using a panel of 44 SNPs spanning the four casein genes, associated variation across *CSN1S1*, *CSN2*, *CSN1S2*, and *CSN3* with protein content, total solids, and milk energy, while *CSN1S2* intron variants tracked milk yield [16,21].

Beyond the caseins, genes controlling lipid metabolism and mammary function have emerged as key determinants of yield and fat composition. *DGAT1*, which encodes a rate-limiting enzyme in triacylglycerol synthesis, is a focal point of milk-production genetics, and several of its polymorphisms associate with production traits in cattle, buffalo, goat, and sheep [22]. Variants in hormonal and lipogenic pathways are similarly implicated: polymorphisms in *ACACA*, *BTN1A1*, *LPL*, and *SCD* associate with daily milk yield, fat and protein percentages, and somatic cell count in Czech dairy-goat breeds, providing information directly usable for marker-assisted selection [16], whereas *PRL*, *DGAT1*, *FSHR*, and *GH* regulate lactation and reproductive performance and thereby constitute priority markers for breeding programs [22,23]. Single-nucleotide polymorphisms, insertions/deletions, and copy-number variants at these loci have been repeatedly linked to differences in production performance across breeds and populations.

**Figure 1:**
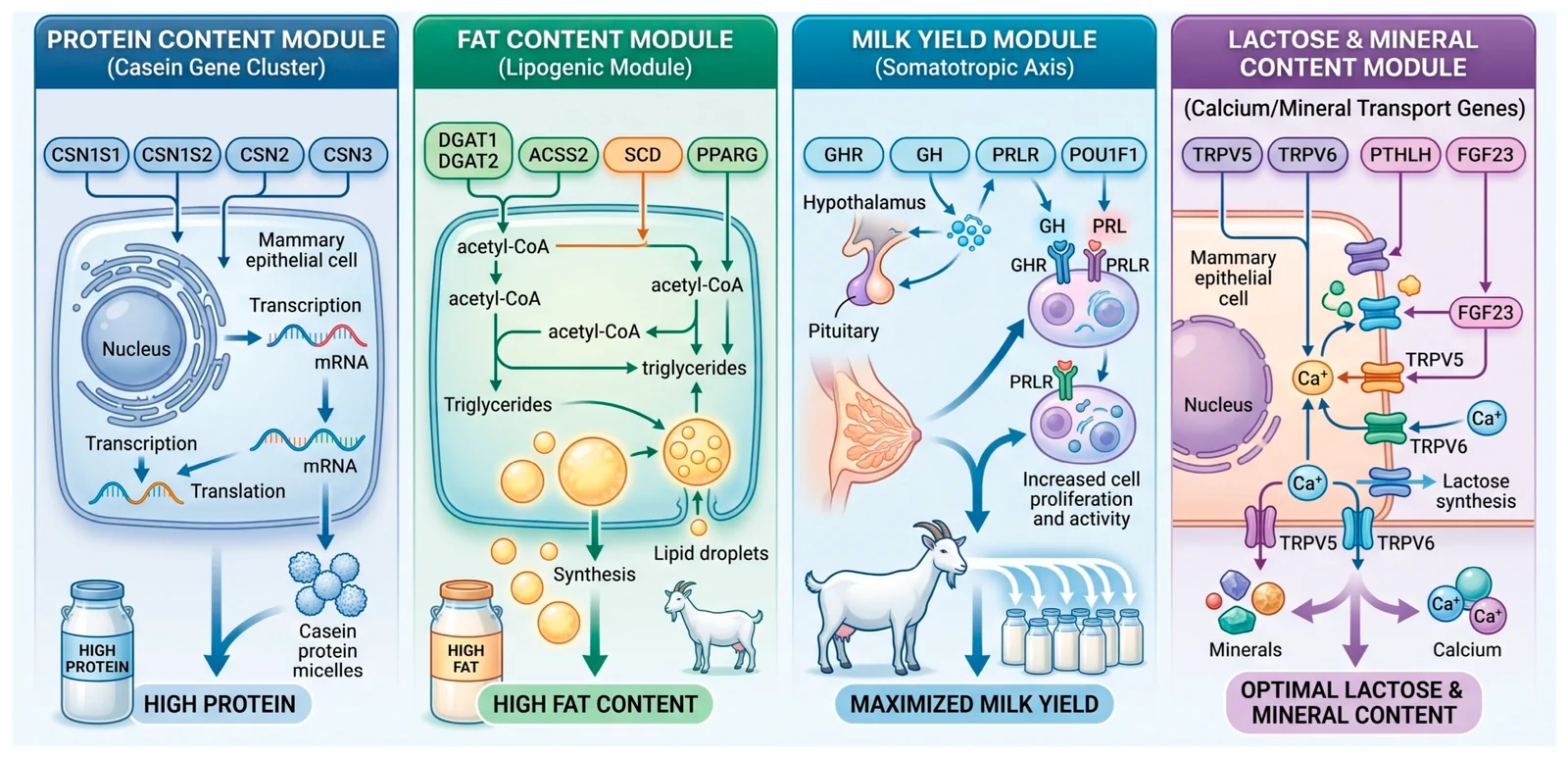
Key genes controlling milk production traits in dairy goats. Candidate genes group into four functional modules, each mainly affecting one trait. The casein gene cluster—*CSN1S1* (αS1-casein), *CSN1S2* (αS2-casein), *CSN2* (β-casein), and *CSN3* (κ-casein)—controls protein content. The lipogenic module—*DGAT1* and *DGAT2* (diacylglycerol O-acyltransferase 1 and 2), *ACSS2* (acyl-CoA synthetase short-chain member 2), *SCD* (stearoyl-CoA desaturase), and *PPARG* (peroxisome proliferator-activated receptor gamma)—controls fat content. The somatotropic axis—GHR (growth hormone receptor), *GH* (growth hormone), *PRL* (prolactin), *PRLR* (prolactin receptor), and *POU1F1* (POU class 1 homeobox 1)—governs milk yield. The calcium-/mineral-transport genes—*TRPV5* and *TRPV6* (transient receptor potential cation channel V5 and V6), *PTHLH* (parathyroid hormone-like hormone), and *FGF23* (fibroblast growth factor 23)—affect lactose and mineral content. Figure created with BioRender.com. Note that the schematic is based on speculative information rather than validated data; the relationships shown should therefore be interpreted with caution.

Despite this progress, reported gene–trait associations frequently diverge among studies and populations, reflecting differences in breed genetic background, allele frequencies, environmental conditions, and analytical strategy [13]. Consolidating this dispersed evidence is therefore essential to identify which genes and polymorphisms are the most reliable determinants of milk production traits and to guide their practical deployment. Accordingly, this review synthesizes current knowledge of the key genes and polymorphisms associated with milk production traits in dairy goats (Figure 1), evaluates the reported effects of these variants on milk yield and composition across whole-genome, association, transcriptomic, and functional studies, and appraises their utility for marker-assisted and genomic selection.

Although multiple GWASs, whole-genome resequencing (WGS) investigations, and transcriptomic analyses have identified numerous such genes and polymorphisms in dairy goats, the findings remain highly scattered across diverse breeds, geographic contexts, analytical platforms, and study designs. These inconsistencies mean that no prior synthesis has systematically evaluated which candidates are truly reproducible across this heterogeneous body of evidence, nor integrated selection-signature, GWAS, transcriptomic, single-cell, and metabolomic layers into a unified, breeding-relevant mechanistic framework. This review fills that gap: it is the first comprehensive multi-omic synthesis focused on 2020–2026 literature that identifies the most reproducible genetic determinants of dairy goat milk yield and composition, resolves the regulatory networks (*SREBP1*, *PPARG*, *ELF5*, and non-coding RNAs) translating genotype into phenotype, and appraises their practical deployment potential—a level of mechanistic integration not provided by any predecessor review on this topic.

This review is directed at three overlapping readerships: (i) animal geneticists, genomic researchers, and molecular biologists seeking a critically appraised synthesis of the current gene-mapping and functional literature in dairy goats; (ii) animal breeders, breeding organization technical officers, and applied geneticists who require guidance on which candidate genes have sufficient reproducibility to merit incorporation into marker-assisted or genomic selection indices; and (iii) graduate students and early-career researchers who need an integrative overview of goat milk genetics. Technical genomic content is presented with full scientific precision, while each major section concludes with a clear summary of practical breeding implications to serve this mixed readership.

## 2. Literature Search Strategy

This narrative review was conducted to synthesize current evidence on the genetic determinants of milk production traits in dairy goats. Four electronic databases—PubMed, Web of Science, Scopus, and Google Scholar—were searched using key terms combined with Boolean operators (AND/OR): “dairy goat,” “milk production traits,” “candidate gene,” “genome-wide association study,” “quantitative trait loci,” “casein,” “fatty acid synthesis,” “genomic selection,” “whole-genome resequencing,” “selection signature,” “transcriptomics,” and “marker-assisted selection.” Reference lists of retrieved articles were also screened to capture additional relevant studies.

The search primarily targeted publications from January 2020 to July 2026. Older studies were included where they provided foundational evidence or essential context not covered by more recent work.

Articles were included if they were peer-reviewed original research, genome-wide association studies, whole-genome resequencing investigations, transcriptomic or multi-omic studies, or meta-analyses reporting on genetic variants or regulatory mechanisms associated with milk yield, fat, protein, or lactose content in dairy goats, and published in English with the full text available. Conference abstracts, editorials, opinion pieces without original data, and non-English articles without full-text availability were excluded.

Titles and abstracts were first screened for relevance, followed by full-text assessment against the above criteria. As this is a narrative review, no formal risk-of-bias assessment or quantitative meta-analytic synthesis was performed. The included studies were critically appraised and organized thematically, and discrepancies among studies were discussed to ensure balanced coverage.

## 3. Whole-Genome Resequencing and Selection Signatures for Milk Production Traits

The domestication of goats and the centuries of directional breeding that followed have collectively sculpted the genome into a mosaic of regions reflecting natural adaptation and deliberate human selection. These selective episodes leave behind molecularly recognizable footprints—local reductions in nucleotide diversity, extended blocks of haplotype homozygosity, and elevated genetic differentiation between populations—each of which serves as an archeological record of past selection pressures [24,25,26,27]. The pronounced phenotypic divergence that now exists between dairy and non-dairy breeds makes goats a particularly tractable system for exploiting these signals, since the genomic contrast between populations subjected to opposing selection regimes amplifies the statistical power to detect functionally relevant loci [28]. Whole-genome resequencing (WGS) has emerged as the methodological cornerstone of this approach, simultaneously resolving population structure and identifying polymorphisms with genuine relevance to lactation performance—a prerequisite for rational improvement of dairy lines.

The most analytically productive WGS designs are those that place dairy and non-dairy populations in direct contrast, thereby exposing genomic regions swept specifically during selection for lactation capacity. Across European, African, and Asian goats, such comparisons have converged on a recurring constellation of milk-associated regions despite differences in breed composition, sample size, and statistical frameworks. Surveys of European commercial breeds identified strong differentiation at *VPS13C*, *NCAM2*, *TMPRSS15*, *CSN3*, and *ABCG2* [29], while a parallel dairy-versus-non-dairy comparison additionally highlighted the lipogenic and growth-axis regulators *GHR*, *DGAT2*, *ELF5*, *GLYCAM1*, *ACSBG2*, and *ACSS2* [30]. That this convergence is substantive rather than coincidental is compellingly supported by the work of Peng et al. [31], who applied five complementary detection statistics—runs of homozygosity (ROH), composite likelihood ratio (CLR), F~ST~, cross-population extended haplotype homozygosity (XP-EHH), and cross-population composite likelihood ratio (XP-CLR)—to a panel of dairy and wild Bezoar goats. *ACSS2* was independently recovered by all five methods and validated as a dairy-specific target through extended haplotype and evolutionary conservation analysis, placing it alongside *DGAT2*, *GHR*, *B4GALT1*, *VPS13C*, *NFX1*, and *PRPF6* as the most robustly supported candidates. The biological pathways enriched across these loci—acyl-CoA metabolism, glycerolipid biosynthesis, galactose metabolism, and growth-hormone signaling—trace a coherent biochemical trajectory from systemic nutrient flux through to the molecular machinery of milk synthesis.

Resequencing studies drawing on Chinese breed diversity have corroborated several of these candidates while simultaneously expanding the catalog. Analysis of dairy goats representing five breeds, augmented by a large body of published genomic data, recovered *MPP7*, *PRPF6*, *DNAJC5*, *TPD52L2*, *HNF4G*, *LAMA3*, *FAM13A*, and *EPHA5* under selection; a companion longitudinal GWAS within the same cohort identified non-synonymous variants in *LAMA3* as being quantitatively associated with milk yield [32]. A separate investigation contrasting native and imported animals further nominated *STK3*, *GHR*, and *PRELID3B*, with allele frequency divergence between populations lending further credibility to these candidates [33]. The repeated emergence of *GHR*, *VPS13C*, and *PRPF6* across studies that differ in breed representation, analytical methodology, and geographic scope constitutes the most compelling available evidence that these loci reflect genuine selective sweeps rather than demographic artifacts. The evolutionary relevance of these signals extends beyond *Capra hircus*: Akhatayeva et al. [34], demonstrated that *CLASP1* carries a convergent selection signature shared between goats and sheep, implying a conserved regulatory function in ruminant lactation; the same study highlighted *PRPF6*, *VPS13C*, *TPD52L2*, *NFX1*, and *B4GALT1* alongside enrichment of U2-spliceosomal and propanoate-metabolism pathways.

Breed-level analyses have refined the architecture of these signals within specific genetic backgrounds. Contrasting high- and low-yield Guanzhong goats through F~ST~ and nucleotide diversity ratios, Ni et al. [35] localized *ANPEP*, *ADRA1A*, and *PRKG1* to swept chromosomal intervals. In Pakistani breeds selected under conditions of thermal stress, signatures of selection at *IGFBP3*, *LPL*, *LEPR*, *TSHR*, and *ACACA* reflect an intersection between thermoregulatory adaptation and lactation physiology (Zhang C et al., [36]), while Ethiopian indigenous goats foregrounded *GLYCAM1* and *SRC* as locally adaptive [37]. The degree to which the detectable signal depends on genetic background is perhaps most sharply illustrated by Demir et al. [38], whose mixed-model analysis of Anatolian breeds identified *ID4* and *CXCR4* as direct-effect loci for lactation yield exclusively in the Honamlı breed, with no comparable associations detected in Hair or Kabakulak goats. This context-dependence serves as an important reminder that selective sweeps are neither universal nor breed-agnostic. Complementing these genome-wide perspectives, targeted studies have linked prolactin variants to yield in crossbred Anglo-Nubian animals [39] and implicated *POU1F1*, *PRLR*, and β-lactoglobulin SNPs in milk trait variation in Kilis goats [40]. Collectively, across all the detection strategies deployed to date, the evidence consistently resolves a lipogenic–somatotropic functional core as the primary genetic substrate of superior dairy performance. The principal findings from these selection-signature investigations are consolidated in Table 1.

The evidence most convincingly supports *ACSS2*, *VPS13C*, *GHR*, and *PRPF6* as genuine dairy-specific selective sweep candidates. Their consistent recovery across multiple independent studies using complementary methods (ROH, XP-EHH, FST, CLR) in geographically diverse populations—European, Asian, and African—distinguishes them from population-specific signals that should not be broadly generalized. The biological coherence of the enriched pathways (acyl-CoA metabolism, glycerolipid biosynthesis, and growth-hormone signaling) lends additional mechanistic credibility. Breed-specific signals such as *ID4*/*CXCR4* in Honamlı goats are scientifically valid but require local validation before incorporation into breed-agnostic selection indices.

## 4. Genome-Wide Association Studies of Milk Yield and Composition

While selection-signature analyses identify genomic regions shaped by historical selection pressure, GWAS complement this approach by establishing direct statistical relationships between individual genotypes and quantitatively measured phenotypes. The practical feasibility of GWAS in dairy goats was unlocked by two parallel developments: the availability of a high-quality chromosome-level reference assembly and the commercialization of species-specific high-density SNP genotyping platforms, most notably the GoatSNP50 and GoatSNP53K BeadChips [41,42]. These resources together enabled the systematic dissection of milk fat, protein, and lactose as tractable quantitative traits.

The foundational GWAS of French Alpine and Saanen goats genotyped on the 50K array established several principles that have guided subsequent research. The analysis identified association signals distributed across an extensive number of genomic regions for multiple milk traits, and most consequentially validated *DGAT1* on chromosome 14 as a fat-content determinant, with exon mutations R251L and R396W shown to measurably reduce milk fat percentage [41]. This result crystallized what later work would repeatedly confirm: milk composition is a deeply polygenic trait architecture in which the casein gene cluster exerts dominant control over protein content while an independent lipogenic module, anchored by *DGAT1*, governs fat deposition.

Subsequent GWAS investigations have systematically broadened and mechanistically deepened these foundational associations. Integration of the GoatSNP50 chip with transcriptomic data in a large Murciano-Granadina cohort implicated *CSN1S1*, *CSN2*, *CSN1S2*, and *CSN3* in protein percentage, and additionally highlighted calcium-transport and calcium–phosphorus regulatory genes—*TRPV5*, *TRPV6*, *PTHLH*, and *FGF23*—coupling protein synthesis to mineral homeostasis at the genomic level [43]. The temporal robustness of the casein signal was formally established by Luigi-Sierra et al. [44], whose longitudinal analysis confirmed the chromosome-6 protein-percentage association across the first three successive lactations, indicating that this genomic region exerts a persistent rather than stage-specific effect on protein deposition throughout productive life.

More recent methodological advances have shifted attention toward the regulatory architecture underlying these associations. Applying random forest variable importance ranking followed by information-theoretic epistasis analysis to the Murciano-Granadina dataset, Khan et al. [45] identified transcription-factor hubs—*DBP*, *HAND1*, *HOXA4*, *PPARA*, and *THAP1*—as shared regulatory nodes across a broad set of milk traits, underscoring that the genetic control of milk composition operates substantially through transcriptional regulatory networks rather than solely through structural coding variation. A complementary multi-model GWAS deploying general linear, mixed, and FarmCPU frameworks on Guanzhong goats further resolved associations for milk traits measured by infrared spectroscopy and flow cytometry, nominating candidates spanning immune function, coagulation, cell-cycle regulation, and extracellular matrix biology [40]. The most granular compositional dissection to date, conducted in Karachai goats on the GoatSNP53K platform, identified functionally distinct sets of candidate loci for protein and β-casein on one hand, and for fat content and fatty-acid profile on the other—with additional loci implicated in broader milk quality attributes—revealing that even within the broad category of milk composition, the genetic architectures governing protein and fat traits are substantially non-overlapping [42].

Caseins account for roughly 80% of goat milk protein, and the four genes encoding them—*CSN1S1*, *CSN1S2*, *CSN2*, and *CSN3*—sit in a single cluster on chromosome 6 whose polymorphisms track protein content, fat percentage, and total solids across populations [46,47]. *CSN1S1* is exceptionally variable, with at least 22 described alleles that constitute a rich resource for marker-assisted selection [47]. These alleles fall into four functional classes defined by αS1-casein output: strong (A, B, C, H, L, M; ~3.5–4.2 g/L), intermediate (E, I; ~1.1–1.6 g/L), weak (F, G; ~0.45–0.6 g/L), and null (O1, O2, N; undetectable) [47]. This quantitative ladder converts genotype directly into predicted protein yield, which is precisely what makes *CSN1S1* so attractive for selection.

Allele frequencies, however, are strongly population-specific and carry an adaptive signature. In native goats from arid southern Tunisia, strong alleles dominated at a combined frequency near 0.79 (B, 0.489; A, 0.267; C, 0.033), while null alleles (N + O1) reached 0.133—higher than in Moroccan populations [47]. The authors linked this distribution to survival under water scarcity and temperatures approaching 47 °C: does secreting protein-rich milk provision their kids for rapid growth before the harsh dry season, tying casein variation to reproductive fitness. A contrasting pattern emerges in Chinese breeds. Surveying the *CSN1S1* O1 null allele in 2319 goats from 11 breeds by allele-specific PCR, Wang et al. [46] found the functional allele fixed or nearly so (0.946–1.000), with the locus in Hardy–Weinberg equilibrium and low polymorphism information content throughout; the null-carrying A0 allele appeared only in Saanen (0.054), Laoshan (0.025), and Guanzhong (0.016) dairy goats and was absent from cashmere and meat types. This near-monomorphism stands against Norwegian Dairy goats, where the O1 allele reaches ~70–73% and materially alters milk composition and technological properties [48,49]—a divergence that underscores how breed history and drift, not selection alone, sculpt allele frequencies and why population-specific characterization is indispensable. Beyond *CSN1S1*, an SNP panel spanning all four casein genes in Sarda goats linked the cluster to protein, total solids, and milk energy, with calcium-sensitive genes associated with lipid content and *CSN1S2* intron variants correlating with yield, pH, NaCl, and somatic cell score [21].

Considered collectively, the converging outputs of GWAS and selection-signature analyses triangulate a consistent gene set centered on the casein cluster, the lipogenic module, the somatotropic axis, and calcium–mineral transport. Among these, the casein locus stands out as the single most reproducible genetic determinant of milk protein content, a consistency that invites closer functional and regulatory examination, as addressed in the following section.

## 5. Transcriptomic and Multi-Omic Dissection of Lactation

Genomic and selection-signature analyses identify the chromosomal addresses of variants associated with dairy performance, but they cannot, by themselves, reveal which genes are transcriptionally engaged as the mammary gland cycles through successive phases of growth, secretion, and involution. Transcriptomics addresses this limitation directly, capturing the dynamic regulatory landscape that converts genomic potential into phenotypic output. Over the past decade, studies ranging from bulk tissue profiling to single-cell resolution have progressively assembled a picture of lactation as an orchestrated molecular program governed by layered regulatory logic—encompassing non-coding RNAs, transcription-factor networks, lipid-sensing nuclear receptors, and cross-pathway signaling cascades—all of which bear on the practical goal of improving milk yield and composition.

Functional and multi-omic data now explain how these variants act. By pairing transcriptomics with GWAS, Guan et al. [43] showed that casein-region genes are co-regulated with calcium-ion transport (TRPV5/6) and calcium–phosphorus metabolism (*PTHLH*, *FGF23*), integrating protein assembly with the mineral phase of milk. Metabolomic profiling adds a downstream readout: milk from does carrying weak or null alleles is enriched in lactose-metabolism substrates, whereas strong-allele milk favors tricarboxylic-acid-cycle intermediates, indicating that αS1-casein genotype reshapes mammary energy partitioning [46,47,50]. At the regulatory level, the transcription factor *ELF5* drives casein synthesis through the JAK2/STAT5 pathway: *ELF5* overexpression promotes proliferation, suppresses apoptosis, and raises αS1-, αS2-, β-, and κ-casein expression, an effect abolished when JAK2/STAT5 is inhibited, positioning *ELF5* as a manipulable node for elevating protein output [51]. The practical corollary is direct—in Norwegian and other populations, *CSN1S1* and *CSN2* haplotypes govern total yield and protein content [49], so selecting strong *CSN1S1* alleles is a defensible route to protein-rich milk wherever those alleles segregate.

Among the non-coding regulatory elements that have attracted particular attention, long non-coding RNAs (lncRNAs) have emerged as consequential modulators of milk fat synthesis. The lncRNA *linc8058* exemplifies this class: highly expressed in the mammary gland during active lactation, it operates as a competitive endogenous RNA by sequestering the microRNA chi-miR-342-5p, thereby relieving post-transcriptional suppression of its target *SCARA5* and channeling downstream activation through the PI3K/AKT signaling axis toward enhanced milk fat secretion [52]. That a single non-coding transcript can exert such a topologically central effect illustrates how lncRNA-mediated buffering of miRNA activity constitutes a functionally important layer of mammary gene regulation, one that operates largely invisibly to genomic scans.

MicroRNAs themselves have an independent and stage-specific role in shaping mammary biology across the full lactation cycle. Longitudinal profiling of the mammary gland from late gestation through the dry period in dairy goats reveals that the miRNA landscape is not static but undergoes substantial compositional reorganization, with distinct expression clusters characterizing each physiological transition [31]. The biological significance of this temporal patterning is not merely descriptive: functional investigation of chi-miR-423-3p demonstrated that it suppresses epithelial proliferation and promotes apoptosis by directly targeting *IGF1R*, thereby attenuating PI3K/AKT pathway activity—a mechanistic logic that positions this miRNA as a critical regulator of the involutional remodeling that follows the cessation of active lactation. The broader implication is that the same signaling pathway—PI3K/AKT—functions as a recurring integration point for both lncRNA and miRNA regulatory inputs, suggesting that the pathway occupies a nodal position in mammary physiology whose sensitivity to upstream non-coding signals merits deeper functional characterization.

At the level of protein-coding gene regulation, transcriptomic dissection has identified several functional hubs that coordinate the cellular transitions defining lactation stages. Profiling Laoshan mammary tissue across the lactation cycle, Ji et al. [53] resolved a regulatory network centered on *CCND1*, *TGFBI*, and *ESR1* as principal hub genes. *CCND1* drives G1/S phase transition to sustain the epithelial proliferative capacity required for lactogenesis, while *TGFBI* moderates apoptotic signaling and extracellular matrix remodeling during involution, and *ESR1* orchestrates estrogen-dependent ductal morphogenesis and alveolar maturation. Auxiliary regulators including *WNT4*, *CSF1*, *MSX2*, and *TBX3* contribute to branching morphogenesis and epithelial differentiation, with PI3K-AKT and Hippo pathway enrichment situating these hubs within a broader signaling architecture that coordinates the gland’s developmental trajectory from lactogenesis to involution. A temporally refined analysis spanning peak lactation through involution extended this catalog to include genes with roles in DNA replication fidelity, cytoskeletal dynamics, ubiquitin-mediated proteolysis, and fatty-acid handling—with *PLA2*, *CPT1*, and *PLD* among those specifically nominated for mammary lipid metabolism—alongside enrichment of pathways governing intermediary carbon metabolism [54]. Crisà et al. [55] further demonstrated that stage-wise transcriptional shifts encompass coordinated changes in amino-acid supply, lipid flux, and calcium homeostasis, reinforcing the view that milk composition is not fixed but dynamically adjusted at the transcriptional level throughout lactation.

A particularly important clarification of the lipogenic regulatory hierarchy has come from perturbation experiments. Overexpression of *PPARG* in goat mammary epithelial cells, with and without the pharmacological agonist rosiglitazone, triggered extensive transcriptional reprogramming and positioned *PPARG* as a master regulator of milk fatty-acid metabolism, with substantial downstream crosstalk to *PPARD* and the nuclear receptor *NR1H3* [56]. This functional relationship between lipid-sensing nuclear receptors and the transcriptional control of milk fat synthesis provides a mechanistic framework that contextualizes the recurrent identification of lipogenic candidates in GWAS and selection scans. Similarly, at the metabolic level, the ketone body-metabolizing enzyme BDH1 has been identified as a negative regulator of lipid synthesis in mammary epithelial cells, with R-BHBA upregulating *BDH1* expression and thereby suppressing triacylglycerol accumulation and lipogenic gene activity—an axis that may be relevant to understanding the metabolic constraints on milk fat production during periods of negative energy balance [57].

The integration of transcriptomic findings with single-cell and metabolomic data has substantially amplified the resolution and translational reach of these observations. The first single-cell RNA-sequencing atlas constructed from goat milk resolved distinct immune and epithelial cell populations whose proportional composition correlated quantitatively with somatic cell count, revealing that high-epithelial-fraction samples display pro-inflammatory signaling through *SELL* and *CXCL* networks alongside upregulated casein gene expression, while low-epithelial-fraction samples carry anti-inflammatory signatures [6]. These findings nominate epithelial cell fraction as a candidate biomarker for milk quality and hypoallergenic potential, bridging transcriptomic cell-type resolution with practically actionable phenotypic endpoints.

At the systems level, metabolome-wide association analysis has opened a complementary window by linking specific genomic loci to the biochemical composition of milk with a granularity unattainable by transcriptomics alone. Applying metabolomic GWAS to an extreme-phenotype design in Xinong Saanen goats, Zhang Z et al. [51] resolved associations between distinct metabolite profiles and loci governing fat, solids-not-fat, protein, and lactose fractions—implicating regulators of JAK–STAT signaling, lipoprotein metabolism, TGF-β pathway activity, and cholesterol biosynthesis as determinants of compositional variation across these trait categories. The convergence of these metabolomic loci with candidates previously identified by transcriptomic and genomic studies reinforces the conclusion that milk composition is governed by an interconnected regulatory network rather than by independently acting genes. Finally, transcriptomic characterization of the bioactive protein complement of goat milk—encompassing lactoferrin (*LTF*), lysozyme (*LYZ*), β-casein (*CSN2*), growth-hormone receptor (*GHR*), butyrophilin (*BTN1A1*), and β-lactoglobulin (*LGB*)—provides the molecular basis for understanding the immunomodulatory and bioactive properties that distinguish goat milk as a functional food substrate [7].

Taken together, these multi-omic investigations sketch a regulatory architecture for lactation that is considerably more complex than the genomic studies alone would suggest. Non-coding RNAs modulate transcription-factor networks; nuclear receptors integrate metabolic signals into lipogenic programs; single-cell heterogeneity shapes bulk phenotypic measurements; and metabolomic variation reflects the cumulative output of all these upstream regulatory layers. What the field has not yet fully accomplished—as the existing literature itself acknowledges—is the translation of this rich mechanistic understanding into tangible genetic improvement tools for goat dairy production. That translational gap between transcriptomic insight and practical breeding application represents one of the most pressing challenges for the field going forward.

The chromosome-6 casein cluster represents the single most reproducible genetic determinant of milk protein content in dairy goats, supported by repeated GWAS identification, longitudinal confirmation across three successive lactations [44], and functional validation through ELF5/JAK2-STAT5 studies [51]. *DGAT1* holds a comparable position for milk fat, validated across multiple breeds, analytical platforms, and functional approaches. Both loci have sufficient evidence to justify immediate inclusion in marker-assisted selection panels. Transcription-factor hubs (*DBP*, *HAND1*, *HOXA4*, *PPARA*, *THAP1*) identified by epistasis analyses represent an important but emerging regulatory layer that warrants functional validation before broad deployment.

## 6. Candidate Genes Associated with Milk Production Phenotypic Traits

### 6.1. Molecular Regulation of Lipid Metabolism in Goat Mammary Epithelial Cells

Goat mammary epithelial cells (GMECs) provide the controlled in vitro system in which candidate regulators of milk-fat synthesis can be tested causally rather than correlatively, and the accumulated work now resolves into a small number of interacting control hubs. *SREBP1* sits at the center. Its output is amplified by *FADS2*, whose overexpression raises triacylglycerol (TAG) and lipid-droplet accumulation by transcriptionally activating *SREBP1* [58], and by the Akt1–mTOR axis, whose activation upregulates *SREBP1* and intracellular TAG while its inhibition suppresses lipogenic genes [59]. Countering this, *FoxO1* represses *SREBP1* through LXR- and SREBP-response elements in its promoter, lowering *FASN*, *ELOVL6*, *SCD1*, *DGAT2*, and *GPAM*; *FoxO1* simultaneously sustains ATGL expression, so its depletion raises lipid droplets while its overexpression reduces them [59,60]. *SCD1* feeds back onto the same hub by regulating *SREBP1* and *PPARG1* to set fatty-acid composition and TAG-synthesis rate [61], and pharmacological *LXRB* activation with T09 drives PUFA synthesis through the *LXRB–SREBP1* network [62]. The PPAR family forms a parallel axis: PPARG governs SCD expression and monounsaturated fatty-acid synthesis [63], while *PPARD* promotes fatty-acid activation, lipid-droplet formation, and secretion to maintain mammary homeostasis [64].

Lipolytic and signaling inputs tune this machinery. *ATGL* is the principal lipolytic switch—its overexpression accelerates lipolysis and lowers droplet content while raising free fatty acids [65], and its inhibition raises TAG and droplets while depressing *HSL*, *FABP3*, *PPARα*, *ADFP*, *BTN1A1*, and *XDH* and elevating the uptake receptor *CD36* [66]. Growth-factor and adipokine signaling feed in through EGF, which stimulates fatty-acid synthesis via PLC-γ1/Akt [67], and through *MST1* and *ADIPOR1*, which modulate GMEC lipid metabolism by distinct routes [68,69]. Two recently characterized brakes complete the signaling layer: *TRIB3* suppresses fatty-acid metabolism by inhibiting p-AKT/PPARG and downregulating *GPAM*, *DGAT1*, and *PLIN1* [70], and *BDH1* acts as a negative regulator that counteracts the inhibitory effect of R-β-hydroxybutyrate on lipogenic gene expression [57].

A dense non-coding and epigenetic layer overlays these pathways. Circular RNAs operate as competing endogenous sponges: circ007071, upregulated more than 12-fold at peak lactation, sequesters miR-103-5p to relieve its repression of *PPARγ* and thereby promotes TAG, cholesterol, and saturated C16:0/C18:0 accumulation [71], while circ003429 sponges miR-199a-3p to stabilize *YAP1* and modulate TAG and unsaturated fatty-acid synthesis [72]. Individual microRNAs exert direct control—miR-27a suppresses TAG accumulation [73], miR-26a/b knockout upregulates *INSIG1* to shift TAG and cholesterol [74], and miR-375, miR-10, miR-26, miR-29, and miR-126 mark distinct developmental stages of the gland [75]. Epigenetic marks add a further tier: exogenous docosahexaenoic acid remodels fatty-acid composition through H3K9 acetylation [76], and *METTL14*-mediated m^6^A modification of *CEBPB* mRNA—read by *YTHDF1* and *YTHDF3* to enhance translation—promotes TAG and cholesterol synthesis and droplet accumulation [77]. The regulators governing lipid metabolism in GMECs are consolidated in Table 2, whose organization by mechanistic layer exposes how transcriptional hubs, lipolytic switches, and non-coding regulators are wired into a single, tunable network.

### 6.2. Candidate-Gene Associations and Prospects for Marker-Assisted Selection

Alongside genome-wide discovery, hypothesis-driven candidate-gene studies have validated individual loci and connected them to defined physiological roles, building the marker panel from which selection programs draw. Energy-metabolism genes feature prominently: polymorphisms in UCP2 associate with milk fat, protein, dry extract, and lactose, and *PPARG* variants with protein, dry extract, and lactose yield in Saanen and Alpine goats [78], while *APOB* genotypes—favorable AA at the HaeIII and SmaI sites—predict longer lactation and higher yield through the gene’s role in lipid transport during lactation [79]. Enzymes of fatty-acid metabolism have been resolved at finer allelic resolution in Czech White Shorthaired and Brown Shorthaired goats, where *ACACA*, *BTN1A1*, *LPL*, and *SCD* polymorphisms correlated with daily milk traits: *LPL* g.300G>A shaped daily yield and fat percentage, LPL g.185G>T affected protein percentage, and SCD variants influenced fat percentage [16].

The somatotropic–lactotropic axis supplies a second cluster. GH variants support marker-assisted breeding in Algarvia goats [80]; *POU1F1* and *IGF-1* expression tracks yield in Damascus goats through the lactotropic *POU1F1* pathway that also governs GH and prolactin secretion [81,82] and prolactin-system genes recur across breeds, with PRLR g.62130C>T associating with yield in Egyptian Zaraibi goats [83] and *PRL*, *PRLR*, *LALBA*, and *IGF-IR* affecting yield and protein composition in Liaoning Cashmere and other populations [84,85]. Structural variation contributes independently: *DGAT1* copy-number loss raises freezing-point depression and solids-not-fat in Chinese dairy goats [86], and a CNV survey nominated *ADAMTS20* and *PAPPA2*—a metalloprotease active in mammary-cell differentiation and an IGF-binding-protein protease, respectively—as yield-associated genes across five breeds [87,88]. Positional candidates *MDM1*, *SCIMP*, and *ZNF232* have been tied to milk volume in Saanen goats, though without functional follow-up [89], and the classical structural determinants of composition—αS1-casein, β-casein, DGAT1, and β-lactoglobulin—remain the genes with the most direct control over milk components [19], with *BTN1A1* and *MFG-E8* additionally linked to fat yield and total solids in Xinong Saanen and Guanzhong goats [90], and *DGAT1* confirmed as a promising source of genetic variability in milk-fat characteristics [91]. These validated candidate genes, together with regulators such as *STAT5A*, *MTHFR*, *SCAP*, *AGPAT6*, and the fatty-acid-composition loci recovered from New Zealand dairy goats (*ALOXE3*, *KIF1C*), are compiled in Table 2 at the end of this section, which merges the in vitro functional regulators and the association-derived candidate genes into a single reference.

The remaining challenge is translation. Genomic information already improves prediction: incorporating genome-wide variation raised the accuracy of estimates for 305-day yield, fat and protein content, and somatic cell score in New Zealand dairy goats [92], and rapid, low-cost genotyping frameworks—for instance AS-PCR with mathematical-expectation estimation for the *CSN1S1* O1 allele—now make population-scale screening of specific variants feasible [46]. Yet several constraints temper the pace of application. Dairy-goat reference populations remain far smaller than their cattle counterparts, which limits statistical power for the many small-effect variants that build polygenic traits; standardized, high-quality phenotypes are scarce, particularly for trace and functional milk components; and most candidate genes still lack functional validation [13,93]. Even well-characterized loci carry caveats—the near-fixation of *CSN1S1* in most Chinese breeds constrains association power for its rare genotypes [46], and small cohorts such as the 45-animal Tunisian sample call for expansion and for parallel analysis of *CSN1S2*, *CSN2*, and *CSN3* before firm recommendations can be made [47]. The productive path forward is integrative. Combining genomics, transcriptomics, proteomics, and metabolomics reconstructs the regulatory networks that any single layer only fragments, and it is through this systems view—resolving how genes, proteins, metabolites, and phenotypes connect—that the field will convert its expanding catalog of candidate genes into precise genomic and marker-assisted selection strategies for goat milk yield and quality [13,46,47,51,94].

**Table 2:** Candidate genes and functional regulators associated with milk production traits in dairy goats.

Gene/Regulator	Full Name	Effect on Milk Production Trait	Breeds/Invitro Model	References
*BTN1A1*	Butyrophilin subfamily 1 member A1	Protein, fat, SCS content	WSH & BSH goats	[16]
*LPL*	Lipoprotein lipase	Protein and fat content (plasma TAG metabolism)		
*SCD*	Stearoyl-CoA desaturase	Protein and fat content		
*VPS13C*	Vacuolar protein sorting 13 homolog C	Milk production (recurrent selection target)	European/Asian goats	[29,34]
*ACSS2*	Acyl-CoA synthetase short-chain family member 2	Lipogenesis/milk fat (dairy-specific selection)	Dairy goats	[30,31]
*LAMA3*	Laminin subunit alpha 3	Milk yield (non-synonymous variant)	Chinese dairy goats	[32]
*GHR*	Growth hormone receptor	Milk yield/lipogenesis	Dairy goats	[30,33]
*ID4*/*CXCR4*	Inhibitor of DNA binding 4/C-X-C chemokine receptor 4	Lactation milk yield	Honamlı goats	[38]
*CSN3*	Casein kappa	Protein content		
*DGAT1*	Diacylglycerol O-acyltransferase 1	Milk fat content	Saanen, Alpine goats	[41]
*PAEP*	Progestagen-associated endometrial protein (β-lactoglobulin)	Protein content	Saanen goats	
*ADRA1A*	Adrenoceptor alpha 1A	Protein/β-casein content	Karachai goats	
*INSIG1*	Insulin-induced gene 1	Fat; long-chain and polyunsaturated FA		[42]
*CSN1S1*	Casein alpha s1 (allelic classes)	αS1-casein output/protein content	Tunisian native; 11 Chinese breeds	[46,47]
*CSN2*	Casein beta	Total yield; protein, fat, FFA, SCC content	Norwegian dairy goats	[49]
*CSN1S1*	Casein alpha s1	Total milk yield; protein content	Norwegian, Saanen, Alpine goats	[41,49]
*ELF5*	E74-like ETS transcription factor 5	αS1/αS2/β/κ-casein synthesis (protein)	Dairy goat mammary cells; JAK2/STAT5 pathway	[51]
*BDH1*	3-hydroxybutyrate dehydrogenase 1	Negative regulator of milk lipid synthesis	Dairy goat mammary cells; counteracts R-BHBA on lipogenic genes	[57]
*FADS2*	Fatty acid desaturase 2	TAG content, lipid-droplet accumulation	Dairy goat mammary cells; transcriptional activation of SREBP1	[58]
*RPL3*	Ribosomal protein L3	Modulates energetic balance during lactation	Saanen goat	[59]
Akt1/mTOR	Protein kinase B/mechanistic target of rapamycin	↑ TAG and lipogenic gene expression	GMEC (in vitro): upregulates SREBP1	
*FoxO1*	Forkhead box O1	Represses de novo FA synthesis; sustains ATGL	GMEC (in vitro): inhibits SREBP1 via LXRE/SRE; ↓ FASN, ELOVL6, SCD1, DGAT2, GPAM	[59,60]
*SCD1*	Stearoyl-CoA desaturase 1	Sets FA composition and TAG-synthesis rate	GMEC (in vitro): regulates SREBP1 and PPARG1	[61]
*LXRB* (+T09)	Liver X receptor beta	↑ PUFA synthesis and lipid content	GMEC (in vitro): LXRB–SREBP1 network	[62]
*PPARD*	Peroxisome proliferator-activated receptor delta	FA activation, droplet formation and secretion	GMEC (in vitro): ruminant mammary homeostasis	[64]
*ATGL*	Adipose triglyceride lipase	Accelerates lipolysis; ↓ droplets, ↑ FFA	GMEC (in vitro): inhibition ↑ TAG; ↓ HSL, FABP3, PPARα, ADFP, BTN1A1, XDH; ↑ CD36	[65,66]
*EGF*	Epidermal growth factor	↑ Fatty-acid synthesis	GMEC (in vitro): PLC-γ1/Akt signaling	[67]
*MST1*/*ADIPOR1*	Macrophage stimulating 1/adiponectin receptor 1	Modulate lipid metabolism	GMEC (in vitro): distinct signaling routes	[68,69]
*TRIB3*	Tribbles pseudokinase 3	Suppresses milk fatty-acid metabolism	Dairy goat mammary cells; inhibits p-AKT/PPARG (↓ GPAM, DGAT1, PLIN1)	[70]
circ007071	Circular RNA 007071	↑ TAG, cholesterol, C16:0/C18:0	GMEC (in vitro): sponges miR-103-5p → ↑ PPARγ	[71]
circ003429	Circular RNA 003429	Modulates TAG and unsaturated FA	GMEC (in vitro): sponges miR-199a-3p → stabilizes YAP1	[72]
miR-27a	microRNA-27a	Suppresses TAG accumulation	GMEC (in vitro): targets lipid-metabolism genes	[73]
miR-26a/b	microRNA-26a/b	Shifts TAG and cholesterol	GMEC (in vitro): knockout ↑ INSIG1	[74]
DHA (dietary)	Docosahexaenoic acid	Remodels FA composition	GMEC (in vitro): H3K9 acetylation	[76]
*METTL14*	Methyltransferase-like 14	Milk fat synthesis	Dairy goat mammary cells; m^6^A on CEBPB read by YTHDF1/3	[77]
*UCP2*	Uncoupling protein 2	Milk fat, protein, dry extract, lactose	Saanen & Alpine goats	[78]
*SLC27A6*	Solute carrier family 27 member 6	Lactose content	Saanen, Alpine goats	[79]
*APOB*	Apolipoprotein B	Lactation length, lactose content, SCS		
*GH*	Growth hormone	Milk yield; protein and fat content	Algarvia goat	[80]
*PPARG*	Peroxisome proliferator-activated receptor gamma	Protein, dry extract, lactose; milk fat yield; ↑ SCD and MUFA synthesis (master FA regulator)	Saanen & Alpine goats; in vitro (GMEC): crosstalk with PPARD and NR1H3	[56,63,78,81]
*IGF-1*	Insulin-like growth factor 1	Lactation milk yield	Damascus goat	[81]
*PRLR*	Prolactin receptor	Milk yield (g.62130C>T)	Egyptian Zaraibi goats	[83]
*DGAT1*	Diacylglycerol O-acyltransferase 1 (CNV)	Solids-not-fat; freezing-point depression	Chinese dairy goats	[86]
*ADAMTS20*	ADAM metallopeptidase w/thrombospondin type 1 motif 20	Mammary-cell differentiation; yield and protein	Alpine, Saanen, LaMancha, Nubian, Toggenburg	[87]
*PAPPA2*	Pappalysin 2	Milk production/yield		
*MDM1*, *ZNF232*, *SCIMP*	Mdm1 nuclear protein	Regulates milk volume	French dairy goats	[89]
*ALOXE3*	Arachidonate lipoxygenase 3	Fat yield, protein yield, SCS	Alpine, Nubian, Saanen, Toggenburg, crossbred	[92]
*KIF1C*	Kinesin family member 1C	Fat yield and protein yield		
*ACACA*	Acetyl-CoA carboxylase alpha	Daily milk yield; protein and fat content	WSH, BSH, Alpine goats	[16,95]
*PRL*	Prolactin	Mammary preparation; lactation; lactose/protein/fat	Alpine, Liaoning Cashmere goats	[84,96]
*AGPAT6*	Glycerol-3-phosphate acyltransferase 4	Milk yield; TAG (fat) and protein	Xinong Saanen, Guanzhong, Alpine, Damascus	[79,97]
*LALBA*	Lactalbumin alpha	Lactose content; renneting property	Sarda breed	[98]
*MTHFR*	Methylenetetrahydrofolate reductase	Milk protein synthesis	Xinong Saanen, Guanzhong goats	[99]
*STAT5A*	Signal transducer and activator of transcription 5A	Milk yield and fat content	Xinong Saanen, Guanzhong goats	[100]
*SCAP*	SREBP cleavage-activating protein	De novo saturated FA; short-chain & unsaturated FA		
*PPARGC1A*	PPARG coactivator 1-alpha	Energetic metabolism, adipogenesis; FA content		
*OLR1*	Oxidized low-density lipoprotein receptor 1	Saturated and unsaturated FA content	Sirohi goat	[101]
*FABP3*	Fatty acid binding protein 3	FA uptake/transport; butyric and arachidic acid		
*MASP1*/*LCORL*	MBL-associated serine protease 1/ligand-dependent corepressor-like	Multiple milk traits	Guanzhong dairy goats	[102]

Note: ↓ represents suppression/down-regulation while ↑ shows up-regulation/overexpression.

### 6.3. Clinical and Translational Significance: Critical Appraisal and Priority Ranking of Candidate Genes

Because this review addresses the genetic architecture of milk production in an animal-breeding context, the endpoint analogous to clinical significance in human medicine is twofold: the value of each variant for genetic-improvement decisions, and its downstream significance for milk-quality and health-related milk attributes relevant to the animal and to the consumer, namely protein and cheese-making yield, hypoallergenic low-αS1 milk, and milk fatty-acid nutritional profile. This section makes that significance explicit and ranks the candidate genes accordingly; Table 3 summarizes, for each priority gene, its clinical and translational significance, the recommended genotyping approach, and its breeding readiness. Not all candidate genes reviewed here are equally ready for practical deployment. We propose a three-tier classification based on evidence strength, cross-population reproducibility, and mechanistic validation:

Tier 1—Strongest Evidence; Immediately Implementable: *CSN1S1* strong-allele genotyping for protein content and hypoallergenic potential; *DGAT1* as a fat-content marker (validated across breeds, methods, and functional studies); and *GHR*/*PRLR* for lactation yield. *CSN1S1* allele-specific PCR is validated at population scale [46] and recommended for routine herd screening. The αS1-casein (*CSN1S1*) major gene is already incorporated into the official French dairy-goat genomic evaluation for protein content through weighted single-step GBLUP, providing a concrete operational template for embedding a major gene in a national breeding scheme [103,104,105]. For fat content, the causal *DGAT1* missense mutations R251L and R396W have been validated in French Saanen and Alpine goats [41] and are strong candidates for the same kind of incorporation, although they are not yet a routine component of the national evaluation [105].

Tier 2—Good Evidence; Near-Term Integration: *CSN2*, *CSN3*, *ACACA*, *LPL*, *SCD*, *ACSS2*, and *VPS13C* have credible evidence from ≥2 independent study designs or populations. Effects are sufficiently population-specific to require local allele-frequency characterization and validation before adoption. Their translational relevance spans cheese technology (*CSN3* curd firmness and renneting), milk-fat nutritional profile (*SCD*, *LPL*), consumer-health positioning (*CSN2* A2-type β-casein), and production efficiency (*ACSS2*, *VPS13C*). Integration into breed-specific genomic reference panels using available GoatSNP50/53K platforms is the recommended next step.

Tier 3—Emerging; Requires Functional Validation: *LAMA3*, *FADS2*, *ELF5*, *METTL14*, and *linc8058* have compelling in vitro functional or transcriptomic evidence but lack large-scale population association validation. *ELF5*—which drives casein synthesis through JAK2/STAT5 [51]—and the *SREBP1–PPARG* axis for fat synthesis [56,62] represent the highest-priority targets for follow-up functional research and longer-term precision gene-editing applications. For each of these loci the appropriate path is functional confirmation followed by association testing before any breeding recommendation.

In terms of consumer-relevant applications: for improved fatty-acid nutritional profile, selection should target *PPARG*, *SCD*, and *FADS2* variants; for reduced allergenicity, selective breeding toward weak or null CSN1S1 alleles must be embedded in a multi-trait index to compensate for the associated reduction in protein yield; for production efficiency, *ACSS2*, *GHR*, and *PRLR* provide tractable genomic targets. The greatest remaining challenge is not candidate gene discovery but the systematic transition from statistical association to functional proof and from proof to practical breeding implementation, a gap that requires coordinated international investment in larger reference populations, standardized phenotyping pipelines, and genomic evaluation models that accommodate the genetic diversity of local dairy goat breeds worldwide.

## 7. Conclusions and Future Directions

The genetic architecture of dairy goat milk production is now resolved in sufficient detail to identify a consistent and actionable core of genetic determinants. Selection-signature analyses repeatedly recover a lipogenic–somatotropic module—*ACSS2*, *DGAT2*, *GHR*, *VPS13C*, and *PRPF6* among the most reproducible—as the primary genomic substrate shaped by domestication and directional selection for lactation capacity. GWASs independently confirm two governing systems: the chromosome-6 casein cluster (*CSN1S1*, *CSN1S2*, *CSN2*, *CSN3*) as the most reproducible determinant of milk protein content, and *DGAT1* as the principal controller of milk fat. Functional dissection in goat mammary epithelial cells has resolved these loci into tunable regulatory networks centered on *SREBP1*, *PPARG*, and *ELF5*, modulated by an extensive non-coding and epigenetic layer encompassing lncRNAs (linc8058), circRNAs (circ007071), microRNAs (miR-27a, miR-26a/b), and m^6^A methylation (*METTL14*). *CSN1S1* remains the paradigmatic selection marker, its graded allelic architecture converting genotype into predicted αS1-casein output, though allele frequencies are strongly population-specific and demand local characterization before broad deployment recommendations can be issued.

At the gene level, dairy goat milk improvement can be pursued through three complementary strategies. First, marker-assisted selection using Tier-1 loci—CSN1S1 strong alleles, *DGAT1* R251L/R396W, and *GHR*/*PRLR* variants—is feasible today using validated, low-cost genotyping platforms [41,46]. Second, genomic selection incorporating genome-wide SNP information has demonstrated superiority over pedigree-based evaluation for 305-day yield, fat, and protein content [92], and should be extended to additional breeds as reference populations expand. Third, longer-term precision approaches targeting *ELF5* (to enhance casein synthesis via JAK2/STAT5) and the SREBP1–PPARG axis (to improve fatty-acid composition) offer transformative potential contingent on regulatory acceptance and in vivo functional validation.

Several priorities require urgent attention. First, reference populations must expand substantially: current dairy-goat genomic reference panels range from 500 to ~3000 animals in the most resourced national programs, yielding GEBV accuracies of 0.30–0.55 for milk traits—substantially below the 0.60–0.80 achievable in Holstein cattle. International data-sharing consortia modeled on EuroGenomics represent the most tractable short-term response. Second, phenotyping must be standardized and deepened; mid-infrared spectroscopy calibration offers cost-effective access to detailed fatty-acid profiles and casein fractions at milk-recording scale and should be adopted as a standard tool. Third, structural variation (*DGAT1* copy-number loss, *ADAMTS20*, *PAPPA2*) must be integrated alongside SNP panels. Fourth, functional validation must close the gap between statistical discovery and mechanistic proof: few of the hundreds of candidate genes identified to date have been validated in vivo.

The practical challenges of implementing genomic selection in dairy goats must not be underestimated. Compared with dairy cattle, important structural limitations include: fragmented breeding programs organized through nationally isolated breed societies with incompatible phenotypic recording systems; limited availability of high-quality data for functional milk components; reduced genomic prediction accuracy across genetically diverse local breeds, because standard GBLUP models assume long-range LD that breaks down across breed boundaries; and genotyping costs that remain prohibitive for smallholder producers in low- and middle-income countries where most of the world’s dairy goats are managed. Bayesian variable-selection approaches (BayesR, BayesB) calibrated in admixed multi-breed reference panels, and low-density SNP imputation anchored to locally constructed reference panels, offer methodological pathways that deserve prioritization in future research and policy investments.

The most productive path forward is explicitly integrative. Single-cell atlases resolving epithelial fraction as a milk-quality biomarker, metabolome-wide association analyses linking metabolite profiles to composition loci, and transcriptome-wide perturbation experiments ranking master regulators (*PPARG*, *ELF5*) each contribute an explanatory layer no single approach can reconstruct alone. Coupling these multi-omic insights with rapid, low-cost genotyping frameworks already validated for casein alleles offers a realistic near-term route to converting mechanistic knowledge into breeding decisions. Realizing the full economic, nutritional, and environmental value of dairy goat production will depend on this systems-level synthesis, sustained by larger well-phenotyped populations, rigorous functional validation to distinguish causal variants from population-specific noise, and the international collaborative structures needed to achieve the scale that individual national programs cannot reach alone.

## Figures and Tables

**Table 1:** Whole-genome resequencing and selection-signature studies identifying candidate genes for milk production traits in goats.

Population/Sample	Method(s)	Candidate Genes for Milk Traits	Reference
140 goats (Asian, African, European)	Comparative population genomics	*VPS13C*, *NCAM2*, *TMPRSS15*, *CSN3*, *ABCG2* (European)	[29]
Dairy vs. non-dairy goat populations	Comparative genomics	*GHR*, *DGAT2*, *ELF5*, *GLYCAM1*, *ACSBG2*, *ACSS2*	[30]
34 dairy + 24 wild Bezoar goats	ROH, CLR, F_st_, XP-EHH, XP-CLR	*ACSS2* (all 5 methods), *DGAT2*, *GHR*, *B4GALT1*, *VPS13C*, *NFX1*, *PRPF6*, *FA2H*, *AMPD1*	[31]
348 dairy goats (5 breeds) + 126 published genomes	SNP/Indel selection signatures; longitudinal GWAS	*MPP7*, *PRPF6*, *TPD52L2*, *HNF4G*, *LAMA3*, *FAM13A*, *EPHA5*	[32]
134 native + imported dairy goats	WGS, population genetics	*STK3*, *GHR*, *PRELID3B*	[33]
Cross-species goat vs. sheep panel	Selection-signature scan	*CLASP1* (convergent), *PRPF6*, *VPS13C*, *TPD52L2*, *NFX1*, *B4GALT1*	[34]
20 Guanzhong goats (high vs. low yield)	F_st_, π-ratio	*ANPEP*, *ADRA1A*, *PRKG1*	[35]
210 goats (26 Pakistani + 184 worldwide)	WGS, PCA/admixture, LD, selection scan	*IGFBP3*, *LPL*, *LEPR*, *TSHR*, *ACACA*	[36]
44 goats (5 Ethiopian populations)	F_st_, selection-signature detection	*GLYCAM1*, *SRC*	[37]
481 goats (3 Anatolian breeds)	Mixed-model GWAS (GEMMA)	*ID4*, *CXCR4* (Honamlı only)	[38]
208 Guanzhong dairy goats	GWAS (GLM, MLM, FarmCPU)	*MASP1*, *LCORL*, *VWF*, *TNFRSF1A*, *SPATA6*, *MAN1C1*, *BRCA2*	[40]

**Table 3:** Clinical, translational, and breeding significance of the priority candidate genes for dairy-goat milk production.

Gene	Trait/Biological Role	Clinical & Translational Significance	Recommended Genotyping Approach	Breeding Readiness & Context
Tier 1—Strongest evidence; immediately implementable				
*CSN1S1*	αS1-casein; protein and casein content, cheese yield	Principal determinant of protein content and cheese-making yield; weak/null alleles lower αS1-casein and reduce allergenicity, providing the genetic basis for hypoallergenic goat-milk products, while strong alleles raise protein output	Allele-specific PCR (AS-PCR), validated at population scale	Immediate; routine herd screening (Norwegian, Saanen, Alpine, Tunisian, Chinese breeds)
*DGAT1*	Rate-limiting enzyme of triacylglycerol synthesis; milk fat content and solids-not-fat	Primary fat-content marker; variants and copy-number changes shift fat percentage, solids-not-fat, and freezing-point depression, with direct relevance to milk payment and product yield	PCR-RFLP/targeted SNP assay for exon variants; CNV assay where copy-number effects apply	Immediate; fat-content marker-assisted and genomic selection (Saanen, Alpine, Chinese dairy goats)
*GHR*/*PRLR*	Growth-hormone receptor/prolactin receptor; lactation yield and lipogenesis	Yield markers on the somatotropic–lactotropic axis; support selection for lactation output and persistency	SNP genotyping on array platforms (e.g., *PRLR* g.62130C>T)	Immediate to near-term; dairy goats generally, *PRLR* validated in Egyptian Zaraibi
Tier 2—Good evidence; near-term integration				
*CSN2*	β-casein; total yield, protein/fat/FFA content	A2-type β-casein of interest for milk digestibility and consumer-health positioning; contributes to protein composition	SNP/AS-PCR across the casein cluster	Near-term; requires local allele-frequency characterization (Norwegian dairy goats)
*CSN3*	κ-casein; protein content, curd firmness/renneting	Determines cheese-processing behavior (curd firmness, renneting), a direct dairy-technology endpoint	PCR-RFLP	Near-term (Saanen, Alpine)
*ACACA*	Acetyl-CoA carboxylase α; de novo fatty-acid synthesis; daily yield, protein and fat	Milk-fat yield and composition	SNP genotyping	Near-term (WSH, BSH, Alpine)
*LPL*	Lipoprotein lipase; plasma-TAG uptake; fat and protein percentage	Milk-fat yield and composition (g.300G>A, g.185G>T)	SNP genotyping	Near-term (WSH, BSH)
*SCD*	Stearoyl-CoA desaturase; MUFA synthesis	Improves milk-fat nutritional profile (MUFA, potential CLA)	SNP genotyping	Near-term (WSH, BSH)
*ACSS2*	Acyl-CoA synthetase short-chain 2; dairy-specific selective sweep	Production efficiency through milk-fat synthesis	Array SNP/resequencing	Near-term (dairy goats)
*VPS13C*	Recurrent selection target; overall milk production	General production capacity	Array SNP	Near-term (European, Asian breeds)
Tier 3—Emerging; requires functional validation				
*LAMA3*	Laminin subunit α3; non-synonymous variant; milk yield	Candidate yield locus; effect size and reproducibility not yet established	Targeted resequencing/SNP	Emerging; needs large-cohort association testing (Chinese dairy goats)
*ELF5*	ETS transcription factor; master regulator of casein synthesis via JAK2/STAT5	High-priority protein-synthesis lever; gene-editing target	Functional confirmation (in vitro/in vivo), then association	Emerging; research-stage
*linc8058*	lncRNA; ceRNA sponging chi-miR-342-5p → *SCARA5* → PI3K/AKT; milk-fat secretion	Regulatory node for milk-fat secretion; transcriptomic/in vitro evidence	Functional confirmation, then association	Emerging; research-stage
*FADS2*	Fatty-acid desaturase 2; activates SREBP1, raises TAG	Potential lever on milk PUFA profile; in vitro evidence only	Functional confirmation, then association testing	Emerging; longer-term precision-editing candidate
*METTL14*	m^6^A methyltransferase; m^6^A of *CEBPB*; milk-fat synthesis	Epitranscriptomic lever on lipogenesis	Functional confirmation, then association	Emerging; research-stage

## Data Availability

No new data were created or analyzed in this study. Data sharing is not applicable to this article.
